# The Impact of Artesunate-Amodiaquine on Schistosoma mansoni Infection among Children Infected by Plasmodium in Rural Area of Lemfu, Kongo Central, Democratic Republic of the Congo

**DOI:** 10.1155/2018/3487183

**Published:** 2018-07-16

**Authors:** Kennedy Makola Mbanzulu, Josué Kikana Zanga, Jean Pierre Kambala Mukendi, Felly Mbaya Ntita, Junior Rika Matangila, Hypolite Mavoko Muhindo, Sylvain Mpoyi Wa Mpoyi, Michel Ntetani Aloni, Roger Wumba

**Affiliations:** ^1^Department of Tropical Medicine, Infectious and Parasitic Diseases, Faculty of Medicine, University of Kinshasa, Kinshasa, Democratic Republic of the Congo; ^2^Department of Parasitology, Institute of Tropical Medicine (NEKKEN), Nagasaki University, 1-12-4 Sakamoto, Nagasaki 852-8523, Japan; ^3^National Program of Neglected Tropical Diseases, Public Health Ministry, Democratic Republic of the Congo; ^4^Department of Pediatrics, Faculty of Medicine, University of Kinshasa, Kinshasa, Democratic Republic of the Congo

## Abstract

**Background:**

Malaria and schistosomiasis remain life-threatening public health problems in sub-Saharan Africa. The infection pattern related to age indicates that preschool and school-age children are at the highest risk of malaria and schistosomiasis. Both parasitic infections, separately or combined, may have negative impacts on the haemoglobin concentration levels. The existing data revealed that artemisinin derivatives commonly used to cure malaria present also in antischistosomal activities. The current study investigated the impact of Artesunate-Amodiaquine (AS-AQ) on schistosomiasis when administered to treat malaria in rural area of Lemfu, DRC.

**Methodology:**

A prospective longitudinal study including 171 coinfected children screened for anaemia,* Schistosoma mansoni*, and* Plasmodium falciparum* infections. The egg reduction rate and haemoglobin concentration were assessed four weeks after the treatment with AS-AQ, of all coinfected children of this series.

**Results:**

One hundred and twenty-five (74.4%) out of 168 coinfected children treated and present during the assessment were found stool negative for S.* mansoni* eggs. Out of 43 (25.6%) children who remained positives, 37 (22%) showed a partial reduction of eggs amount, and no reduction was noted in 3.6% of coinfected. The mean of haemoglobin concentration and the prevalence of anaemia were, respectively, 10.74±1.5g/dl , 11.2±1.3g/dl, and 64.8%, 51.8%, respectively, before and after treatment, p<0.001.

**Conclusion:**

The AS-AQ commonly used against Plasmodium allowed curing* S. mansoni* in coinfected children and increasing the Hb level. For the future, the randomized and multicentric clinical trials are needed for a better understanding of the effectiveness of AS-AQ against* Schistosoma spp*. The trial registration number was 3487183.

## 1. Introduction

Schistosomiasis is a parasitic disease world widely distributed, of which the most of the disease burden occurs in Africa [1]. The mass chemotherapy using praziquantel (PZQ) remains the main pillar for schistosomiasis control strategy. This chemotherapy is aimed to reduce the current infection and prevent the development of serious illnesses in specific risk groups, mainly in school-aged children, and it should be repeated over time according to the prevalence of the infection in different foci [1, 2]. According to WHO data in 2013, 261 million people at least needed a preventive treatment against schistosomiasis. However, more than 40 million people have been treated against schistosomiasis in 2013. It is estimated that 90% of people who need treatment against schistosomiasis are found in Africa [1].

The Democratic Republic of the Congo (DRC) is identified as schistosomiasis endemic country, where this parasite is present in almost all the provinces for over a century [3]. In Kongo central province, several foci, Lemfu, Kimpese, Songololo, Nsona-Mpangu, and Kuimba have been described as endemics for either* S. mansoni* or* S. haematobium* [4–7]. On the other hand, DRC is ranged among Africa countries facing with the highest burden of malaria [8]. The planning and implementation of a malaria control program should be based on the epidemiological analysis and implementation of interventions suited to each location. Since 2005, in regard to the dramatic progression of* P. falciparum *drug resistance, DRC has adopted the use of the Artemisin-based combination therapy with AS-AQ as the first-line treatment drug for uncomplicated malaria, while Artemether-Lumefantrine is being used too [9]. Nowadays, it has been reported from studies carried out in other countries the emergence of PZQ-resistant strains of Schistosoma [10]. Thus different findings from studies aiming at finding new alternative drugs, which can serve in the control of schistosomiasis, showed convincing antischistosomal activity of antimalarial including amodiaquine, mefloquine, primaquine, and artemisinin derivatives both in vitro and in vivo [11–18]. Despite the fact that the Ministry of Health has adopted a national plan to control neglected tropical diseases (NTDs), including schistosomiasis, based on mass drugs distributions in schools, whose implementation is progressive in the country since 2012 [19, 20], contrarily from Kinshasa province, in Kongo central province, western part of DRC, the prevalence of schistosomiasis keeps increasing [3]. Nevertheless, the current situation about Schistosoma resistance to PZQ remains unknown. This pilot study is proposed to assess the additional benefit of AS-AQ on schistosomiasis when administered to cure malaria in individuals coinfected with* S. mansoni* in rural area of Lemfu.

## 2. Methodology 

### 2.1. Study Area and Population

The study was conducted among children aged from 3 to 15 years, living at Lemfu, a* S. mansoni* endemic area. Lemfu is located in Kisantu Health Zone, in Kongo central province, western part of DRC, at 150 km from Kinshasa, the capital city of DRC.

### 2.2. Study Design

A prospective longitudinal, noncomparative, and nonrandomized study was conducted among the coinfected children, with a followup period of one month from July to August 2015. The included children have been screened previously for* Plasmodium* and* S. mansoni *infection, respectively, by microscopic examination of Giemsa-stained blood smears, and Kato-Katz technique for stool samples analysis during the study aimed to investigate the prevalence and comorbidity of both parasites. To determine the impact of Artesunate-Amodiaquine on* S. mansoni* infection, the coinfected children were treated according to the national recommended guidelines for the treatment of uncomplicated malaria [9]. In addition the haemoglobin concentration level from each study participant was determined at the beginning and at the end of the followup period.

### 2.3. Inclusion Criteria

Exclusion criteria included a history of malaria infection according to WHO criteria, adverse reaction to artemisin derivatives, women with known or suspected pregnancy, and children who were absent during the study followup.

Exclusion criteria included a history of blood transfusion, a current crisis or history of sickle cell crisis, or acute illness two months prior to the study. Pregnancy status was determined by details from the patient's history and/or by a positive pregnancy test. We excluded from the study all children who were absent during the results assessment.

### 2.4. Detection of Schistosoma Infection Control after Treatment

Stool samples were examined for the presence of Schistosoma eggs using the Kato-Katz method four weeks after the treatment with AS-AQ. To improve the sensitivity of the Kato-Katz technique, three stool samples were collected during three consecutive days from each participant after the treatment with AS-AQ has been administered. The* S. mansoni* eggs from each stool sample were counted during microscopic examination and the number obtained was multiplied by the factor 24 in order to get the number of eggs per gram of feces (epg). Finally, from each individual we considered the average of egg counted for three stool samples. The infection intensities were defined following the guidelines established by WHO into light, moderate, or heavy infections, respectively, as follows: 1–99 epg, 100–399 epg, and >400 epg [21].

Haemoglobin (Hb) levels were determined using a Hemocue^®^ Hb 201 (HemoCue AB, Angelhome, Sweden). Anaemia was defined by a haemoglobin concentration according to the age and sex of child as follows: <11, 11.5,12, and 13g/dl, respectively, for children of less than 5 years, 5–11.9 years, and 12–14.9 children, unpregnant women > 15 years, and men > 15 years. The anaemia was classified as severe (Hb=7 g/dl), moderate (Hb= 7–9.9 g/dl), and mild anaemia (Hb=10– 11.4 g/dl) [22].

### 2.5. Malaria Treatment

The coinfected children received a fixed oral dose of AQ-AS ( 4 mg/kg/ AS 10 mg/kg/ AQ) daily for three consecutive days according to the national treatment guidelines [9]. The drugs used for the study were provided for free by SANRU ASBL to the Health Zone office (Artesunate, Ipca laboratories Ltd, India).

The medicines administration was performed by a nurse of the study team, under direct observation of a physician. The treated children were kept at study site for 30 minutes prior to be released. In the case of vomiting, a dose was repeated during the observation period.

### 2.6. Ethical Considerations

Signed informed consent for participation was obtained in French or in the local language of all participants' guardians or parents. Ethical clearance for the study was obtained from the Ethics Committee of the Kinshasa School of Public Health (approval number: ESP/ CE 077/2015).

### 2.7. Data Analysis

Data were entered into Microsoft Office Excel 2007 spreadsheets and analyzed using Epi Info version 7 and SPSS software version 21 (SPSS IBM, Chicago, Ill, USA). The frequencies and percentages were calculated for categorical variables, paired t-test, and Wilcoxon test were used for statistical analysis of continuous data, according to the variable distribution. The a priori statistical significance level was 0.05. The primary efficacy endpoint was the number of participants cured 28 days after treatment. Treatment outcome was expressed as cure for participants whose status changed from S.* mansoni* egg-positive to negative after treatment and noncure or those whose status remained S.* mansoni* egg-positive after treatment. The rate of cure after treatment was calculated as the percentage of individuals who tested negative for S.* mansoni* eggs after treatment. Secondary, the gained Hb concentration level was assessed by comparing the mean and the prevalence of anaemia before and after treatment.

## 3. Results

### 3.1. General Characteristics of Study Participants (Table 1)

**Figure 1:**
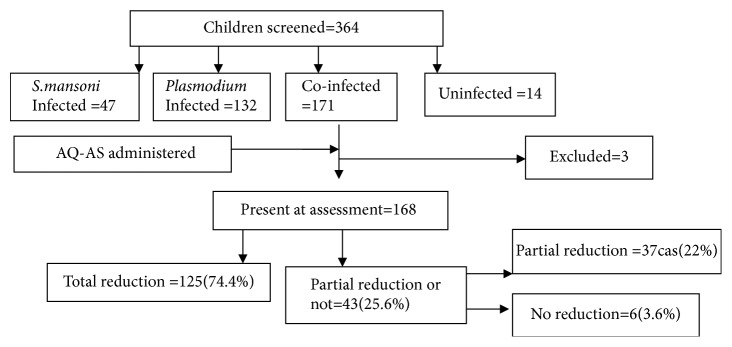
**Summary of baseline study data. **PD: parasitic density; Hb: haemoglobin.

Among the 364 children screened for both parasites, 171 were coinfected with* Plasmodium* and* S. mansoni*. Three were excluded from the study because of their absence at the assessment moment; then 168 were included in the current study (Figure 1). The age of children ranged from 3 to 15 years with mean age of 7.7 years (SD=3.2 years). Males represented 54.9%. The malaria parasite density ranged from 40 to 34000 trophozoites /*μ*l. The number of epg ranged from 24 to 8760 with a median of 192 (IQR: 60–612). The* S. mansoni* infection was, respectively, light (37.6 %), moderate (28.9 %), and severe (33.5%). The overall prevalence of anaemia was 64.6%; its intensity was, respectively, light (56.2%), moderate (42.1%), and severe (1.7%). The mean level of Hb in the study population was 10.8±1.4 g/dl (range: 3.8- 15.5).

**Figure 2:**
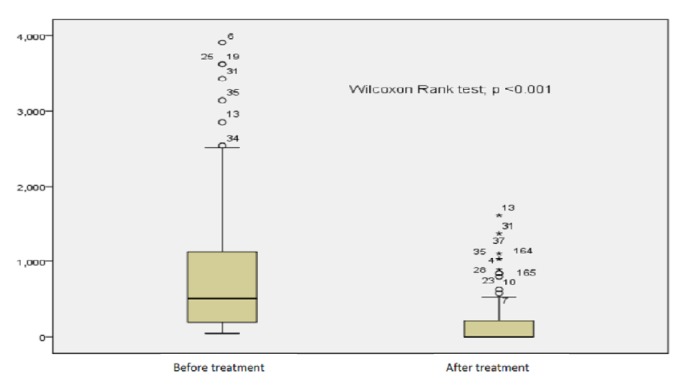
The median egg loads before and after treatment.

Assessment of* S. mansoni* infection and haemoglobin concentration level was performed four weeks after the treatment (Figure 2).

**Figure 3:**
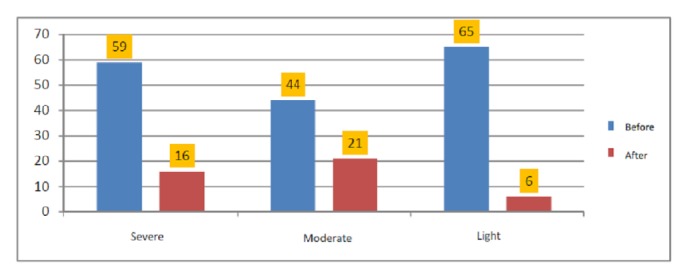
Comparison of* S. mansoni* infection intensities before and after treatment.

Out of all 168 coinfected children,* S. mansoni* infection remained positive in 43 participants (25.6%), of which 37 (22%, 95% CI: 16-29) children had partial reduction of eggs load; and the eggs load tended to increase in 6 children (3.6%) (95% CI: 1.32-7.6). An absence of eggs was observed in 125 children (74.4%) (Figure 1). The median eggs load before treatment was of 504 epg (IQR 48-3912) and zero epg after treatment (IQR: 0-1608) with p<0.001(Figure 2). A considerable change was observed in the* S. mansoni* infection intensity pattern after treatment.  Figure 3 shows the comparison of the infection features before and after treatment.

The prevalence of anaemia passed from 64.8% to 51.8% (p<0.001) and the mean of Hb from 10.74± 1.5g/dl to 11.26±1.3g/dl (p<0.001) after treatment (Table 1).

## 4. Discussion

*Plasmodium *spp. and* Schistosoma *spp. coexist in many regions, and the prevalence of their coinfection varies according to the species of Schistosoma and the geographical location [23–27]; therefore it is highly present in rural health area of Lemfu.

Despite some cases of treatment failures with ACT against malaria reported in different provinces across DRC [27–29], data from multicentric studies have shown that the AS-AQ is effective against malaria in DRC [28–31]. This common therapy combination, widely used across the whole country according to the national policy strategy against malaria, showed an additional benefit on schistosomiasis at the dose administered to cure malaria in coinfected children in rural heath area of Lemfu. The mechanism on how artemisinin affects the Schistosoma parasite remains unknown. However, it was demonstrated by scanning electron microscopy that the tegmentum of the* Schistosoma* was damaged [32] and this effect lasts a few days. The overall cure rate of schistosomiasis in the present study was 74.4%; these findings corroborate with those found elsewhere by previous studies [11–13]. Indeed, in Senegal, Boulanger et al. reported a cure rate of 94.4 % after using AS-AQ in children infected with* S. haematobium *[14], while Keiser et al. found a low cure rate of 21% using AS alone [15]. In Mali, Sissoko et al. observed that* S. hematobium* egg reduction rates were 95.6% with PZQ compared to 92.8% with AS-SMP (p = 0.096)[33].

In earlier study from Kenya, Obonyo et al. reported 65% of cure rate of* S. mansoni *infection in group treated with PZQ compared to 14% found in group treated with AS-SMP (p<0·0001). Later, after changing dose regimen, these authors found no difference in response between the two drugs, and 74% were cured in the PZQ group and 64% in the AS-SMP group (p = 0.4) [34].

Moreover from studies carried out in Sudan and Ethiopia, the authors found 100% of cure rate after using Artemether-Lumefantrine to cure* S. mansoni* infection [16, 17].

The difference in cure rate from those studies could be explained by the different treatment regimens administered, differences in the susceptibility of the parasite to the used medicine, rates of reinfection, and different severity of the infection according to the different geographic locations. In general, it has been reported that artemisinin acts better enough in light infections [16, 17], whereas PZQ showed the highest efficacy against adult parasites, while artemether and artesunate were found to be more effective against juvenile forms preventing them from evolving into adult worms with egg-laying ability. This suggests that a combination of PZQ with artemisinin derivatives might be more effective against schistosomiasis.

Although this study has provided the useful data, budget constraints related to lack of adequate funding did not permit assessing malaria parasitaemia after the treatment of included children. However, other benefits of the treatment with AS-AQ were found as the clinical status of study participants improved after the treatment, as showed by the significant increase of the Hb level, therefore a significant decrease of anaemia in the study population. The use of antimalarials in deworming programs should be eyed with caution, as this could accelerate resistance to these drugs. Nevertheless, since malaria and* Schistosoma* infections largely overlap geographically, the wide use of antimalarials might be beneficial in coinfected patients [32]. In the future, multicentric, randomized clinical trial studies using different types of ACT, alone or combined with the PZQ and compared to PZQ will provide useful information on PZQ effectiveness against* Schistosoma* in Lemfu as well as in DRC and likely a new alternative therapeutic against schistosomiasis.

## 5. Conclusion

The present study has shown promising results about AS-AQ in the treatment of* S. mansoni* infection. This is of a capital importance in malaria and schistosomiasis endemic settlements. It is advantageous for individuals harboring Schistosoma parasites, as every time they will be treated with an ACT for malaria, they will be indirectly treated against schistosomiasis and its morbidity. Nevertheless, the heavy burden of the morbidity due to schistosomiasis is implying an integrated approach on pharmacological approaches, population-based chemotherapy, treatment of individual cases; the control of snails, intermediate hosts, with molluscicides, and improving the health education, behavioral changes, sanitation, and safe water supply.

## Figures and Tables

**Table 1:** General characteristics of study participants.

Variables	n (%)	IC 95%	**Mean ±SD**	**Median (IQ)**	**Range**
Total effectif	364				
Weight (Kg)				18.5 (15.5-23)	9-54
**Age (years)**			7.7±3.2		3-15
**Sex**					
Female	164 (45.1)	39.8-50.3			
Male	200 (54.9)	49.6-60.1			
**Age groups (years)**					
3 - 5	120 (33.0)	28.2-38.1			
6-9	140 (38.5)	33.4-43.6			
10-15	104 (28.5)	24.-33.6			
**School level of parents/guardians**					
No instruction	8 (2.2)	1.0-4.4			
Primary	140 (38.7)	33.7-43.9			
Secondary	209 (57.7)	52.5-62.8			
University	5 (1.4)	0.5-3.3			
**Profession of parents/guardians**					
Mechanic	1 (0.3)	0.01-1.6			
Agriculture	351 (96.4)	93.8-98.0			
Teaching	9 (2.5)	1,21-4,81			
Commerce	3 (0.8)	0.2-2.5			
**Taking praziquantel during last campaign**	24 (6.6)	4.3-9.8			
**Histories of symptoms two weeks before and clinic signs**					
History of fever	180(49.4)	44.2-54.7			
Abdominal pain	134(36.8)	31.8-42.0			
Melaena	117(32.1)	27.4-37.2			
Headache	85(23.3)	19.2-28.1			
Diarrhea	42(11.5)	8.5-15.3			
Vomiting	27(7.4)	5.0-10.7			
Anorexia	3(0.8)	0.2-2.6			
Haematuria	2(0.5)	0.1-2.2			
Cough	5(1.3)	2.0-6.1			
Vertigo	1(0.2)	0.01-1.7			
Mictalgie	9(2.4)	1.2-4.8			
Skin rash	13(3.5)	2.0-6.1			
Hepatomegaly	132(36.5)	31.3-41.4			
Splenomegaly	152(41.7)	36.6-47.0			
**Use of bednet last night**	64 (17.6)	13.8-21.9			
**Parasitic infections**					
*Plasmodium *spp.	303(83.2)	78.9-86.8			
*S. mansoni*	218(59.8)	54.6-64.9			
*A. lumbricoides*	8(2.2)	1.0-4.4			
*T. trichuris*	5(1.3)	0.5-3.3			
Co-infection malaria + Schistosomiasis	171(46.9)	41.6-52.2			
Malaria PD(trophozoites//*µ*l)				380(320-800)	40-34000
*S. mansoni* eggs load(eggs /g of feces)				192(60-612)	24-8760
***S. mansoni* infection intensities**					
Light	82(37.6)	31.1-44.4			
Moderate	63(28.9)	22.9-35.4			
Severe	73(33.5)	27.2-40.1			
**Anaemia **	235(64.6)	59.3-69.4			
**Anaemia intensities**					
Light	132(56.2)	49.5-62.6			
Moderate	99(42.1)	35.7-48.7			
Severe	4(1.7)	0.4-4.3			
**Hb (g/dl)**			10.8±1.4		3.8-15.5
